# Early life cognitive development trajectories and intelligence quotient in middle childhood and early adolescence in rural western China

**DOI:** 10.1038/s41598-019-54755-1

**Published:** 2019-12-04

**Authors:** Zhonghai Zhu, Suying Chang, Yue Cheng, Qi Qi, Shaoru Li, Mohamed Elhoumed, Hong Yan, Michael J. Dibley, Wafaie W. Fawzi, Lingxia Zeng, Christopher R. Sudfeld

**Affiliations:** 1 Department of Epidemiology and Biostatistics, School of Public Health, Xi’an Jiaotong University Health Science Center, Xi’an, Shaanxi 710061 P.R. China; 2 Department of Global Health and Population, Harvard T.H. Chan School of Public Health, Boston, MA USA; 3 United Nations Children’s Fund, China Office, Beijing, 100600 P.R. China; 4 Department of Nutrition and Food Safety Research, School of Public Health, Xi’an Jiaotong University Health Science Center, Xi’an, Shaanxi 710061 P.R. China; 5 Nutrition and Food Safety Engineering Research Center of Shaanxi Province, Xi’an, 710061 Shaanxi China; 6 Key Laboratory of Environment and Genes Related to Diseases, Xi’an Jiaotong University, Ministry of Education, Xi’an, 710061 Shaanxi China; 7 School of Public Health, University of Sydney, Sydney, New South Wales Australia

**Keywords:** Epidemiology, Paediatric research

## Abstract

The relationship of cognitive developmental trajectories during the dynamic first years with later life development outcomes remains unclear in low- and middle-income countries. 1388 Children born to women who participated in a randomized trial of antenatal micronutrient supplementation in rural China were prospectively followed. Cognitive development was assessed six times between 3 and 30 months of age using Bayley Scales of Infant Development, and then in mid-childhood (7–9 years) and early adolescence (10–12 years) using Wechsler Intelligence Scale for Children. We identified four distinct infant cognitive development trajectory subgroups using group-based trajectory modeling: (i) consistently above average, (ii) consistently average, (iii) started below average and then improved, and (iv) started below average and then declined. LBW infants (<2500 g) were 10.60 times (95% CI 3.57, 31.49) more likely to be in the trajectory group that started below average and then declined, while each grade increase in maternal education decreased the risk of being in this group by 73% (95% CI 54%, 84%). Infants who performed consistently above average had 8.02 (95% CI 1.46, 14.59) points higher IQ in adolescence versus the declining trajectory group. These findings suggest that interventions to improve early child development trajectories may produce long-term human capital benefits.

## Introduction

An estimated 250 million children under five years living in low- and middle- income countries (LMICs) failed to reach their full developmental potential^1^. Adversities and risk factors during the first 1000 days of life lay the foundation for development and have long-term consequences across the lifecourse^1–4^. Studies have shown that suboptimal childhood cognitive development is associated with higher risk of coronary heart disease, reduced human capital, and increased risk of mortality and poor health outcomes later in life^5–8^.

A number of modifiable risk factors for suboptimal development have been identified including poverty-related factors, inadequate stimulation, environmental and nutritional factors^9,10^. Nevertheless, a limitation of studies that examined early life determinants of cognitive development typically only assess children at a single time-point, which does not capture the dynamic process of child development. In fact, only a few studies from high-income countries focused on preterm infants have assessed cognitive development trajectories in early childhood. One cohort study from UK and Ireland among 315 extremely preterm births found that impaired cognitive trajectory in infancy persisted into early adulthood and there was no evidence of catch-up^11^. In contrast, a study in the US reported catch-up language trajectory from 3 to 12 years among very preterm infants^12^. To the best of our knowledge, no studies have examined the relationship of early child development trajectories with later life development outcomes among the general population in LMICs.

In this study, we used data from a rural Chinese birth cohort, in which the cognitive development was assessed at 3, 6, 12, 18, and 24 months during the first two years of life and then at 30 months, middle childhood (7–9 years) and in adolescence (10–12 years). The main aims of our analysis were to (1) identify distinct trajectories of cognitive development during the first two years of life, (2) examine predictors associated with these trajectories, and (3) assess whether these trajectories were associated with long-term cognitive outcomes in middle childhood and early adolescence.

## Results

A total of 1388 children were included in group-based trajectory modelling (GBTM) analyses. Baseline characteristics of these participants are presented in Table 1. A total of 669 and 735 of these participants were followed at middle childhood (7–9 years) and early adolescence (10–12 years), respectively (Supplementary Fig. S1). The mean age at middle childhood and adolescence were 7.8 years (SD ± 0.6) and 11.3 (SD ± 0.6) years, respectively. Most background characteristics were similar between individuals who completed the middle childhood and early adolescence assessments and those who were lost to follow-up (Supplementary Table S1).

**Table 1:** Background characteristics of infants included in group-based trajectory analysis in a Chinese birth cohort study (N = 1388)^a^.

Factors	No. (%)/Mean (SD)	Factors	No. (%)/Mean (SD)
Maternal age	24.4(4.4)	Child characteristics	
Maternal education		Male	845(60.9)
<3 years	61(4.4)	Birth weight (g)	3185(410)
Primary	341(24.6)	Weeks of gestation at birth	39.9(1.6)
Secondary	798(57.6)	Preterm (<37 weeks gestation)	50(3.6)
High school+	185(13.4)	Low birth weight (<2500 g)	50(3.7)
Maternal occupation		Small for gestational age(<10^th^ percentile)	165(12.3)
Farmer	1172(84.8)	Age at middle childhood/years	
Others	210(15.2)	Mean (SD)	7.8(0.6)
Paternal age (years)	27.7(4.2)	Range	7–9
Paternal education		Age at adolescence/years	
<3 years	12(0.9)	Mean (SD)	11.3(0.6)
Primary	139(10.1)	Range	10–12
Secondary	914(66.1)	MDI at 30 months of age	86.7(18.7)
High school+	317(22.9)	WISC-IV at school age(7–9 years)	
Paternal occupation		FSIQ	89.5(12.6)
Farmer	1054(76.1)	VCI	87.4(15.5)
Others	332(24.0)	WMI	91.2(12.0)
Household wealth at enrollment		PRI	93.6(13.3)
Low	324(23.3)	PSI	95.3(12.9)
Medium	562(40.5)	WISC-IV at adolescence(10–12 years)	
High	502(36.2)	FSIQ	98.1(12.5)
Parity at enrollment		VCI	102.9(15.6)
0	943(67.9)	WMI	94.5(11.0)
≥1	445(32.1)	PRI	96.0(12.5)
Maternal MUAC (cm)		PSI	100.0(13.7)
<21.5	219(15.9)		
≥21.5	1159(84.1)		
Trial treatment			
Folic acid	496(35.7)		
Iron/folic acid	467(33.7)		
Multiple micronutrients	425(30.6)		

^a^Data are missing for maternal age (n = 2), maternal education (n = 3), maternal occupation(n = 6), paternal age(n = 2), paternal education(n = 6), paternal occupation(n = 2), maternal MUAC(n = 10), birth weight(n = 21), preterm(n = 21), and SGA(n = 51).

Abbreviation: VCI, verbal comprehension index; FSIQ, full-scale intelligence quotient derived from WISC-IV, Wechsler Intelligence Scale for Children-Fourth Edition; MDI, mental development index; MUAC, mid-upper arm circumference; PRI, perceptual reasoning index; PSI, processing speed index; SD, standard deviation; SGA, small for gestational age; WMI, working memory index.

### Identification of child development trajectories during the first two years of life

**Figure 1:**
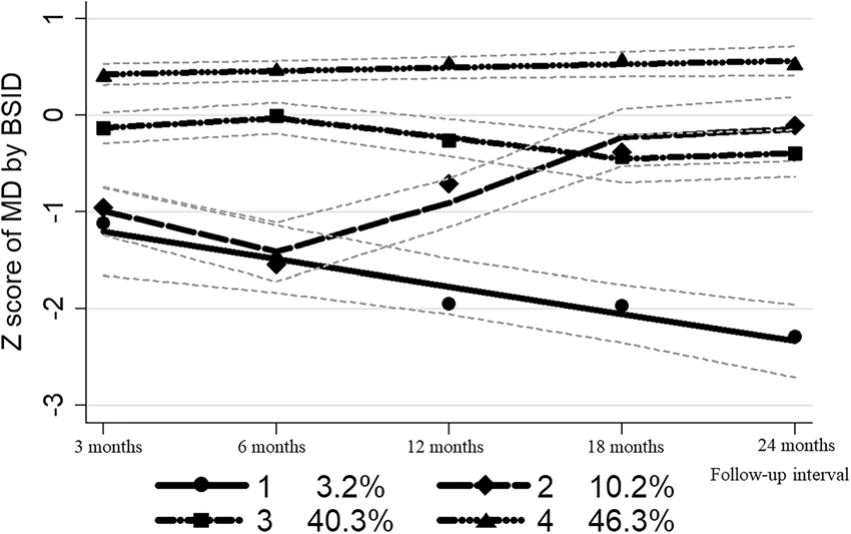
Trajectories of cognitive development identified in infants from 3 to 24 months of age in a Chinese birth cohort study (n = 1388). Lines show for each trajectory the predicated means of z score and 95% confidence limits. Size (%) of each trajectory estimated by the model are presented below the x-axis, which was slightly different from the actual size used in subsequent analysis. The Subgroup 1, 2, 3 and 4 were labeled “Start below average-then decrease”, “Start below average-then increase”, “Consistently average”, and “Consistently above average”, respectively. Abbreviation: MD, mental development; BSID, Bayley Scales of Infant and Toddler Development.

GBTM identified four trajectory subgroups: (1) “Subgroup 1: Start below average-then decrease” (3.2% of all participants), (2) “Subgroup 2: Start below average-then increase” (10.2%), (3) “Subgroup 3: Consistently average” (40.3%), (4) “Subgroup 4: Consistently above average” (46.3%). The fit indexes are presented in Supplementary Table S2 and Fig. 1 graphically presents the final trajectories of cognitive test z scores during the first two years of life.

### Predictors of child development trajectory group

We then compared the distribution of socioeconomic, pregnancy and birth outcome characteristics between the trajectory groups (Supplementary Table S3 and Table 2).We determined that children born to mothers with increasing educational level were less likely to be in the groups that started below average and then declined (RR 0.27, 95% CI 0.16, 0.46), started below average and then improved (RR 0.59, 95% CI 0.41, 0.85) and that performed consistently average (RR 0.76, 95% CI 0.61, 0.94) than being in the group that performed consistently above average. Greater than 180 days of multiple micronutrients supplementation relative to folic acid or folic acid plus iron supplementation <180 days during pregnancy was associated with decreased risk of being in group of children who started below average and then improved (RR 0.37, 95% CI 0.16, 0.90) and group of children who performed consistently average (RR 0.63, 95% CI 0.44, 0.89) compared to the group of children performing consistently above average. Infants who were born low birth weight were 10.60 (95% CI 3.57, 31.49) times more likely to be in group of children who started below and then declined as compared to those who performed consistently above average.

**Table 2:** Multivariate multinomial logistic regression analyses identifying predictors for being in the consistently above average group versus the other development trajectory groups^a^.

Factors	Subgroup 1: Start below average-then decrease		Subgroup 2: Start below average-then increase		Subgroup 3: Consistently average	
	RR (95% CI)	P values	RR (95% CI)	P values	RR (95% CI)	P values
Maternal education per grade increase^b^	0.27(0.16, 0.46)	<0.001	0.59(0.41, 0.85)	0.01	0.76(0.61, 0.94)	0.01
Maternal occupation-non-farmer	0.16(0.02, 1.39)	0.10	0.78(0.28, 2.12)	0.62	0.74(0.46, 1.19)	0.27
Paternal education per grade increase^b^	1.33(0.69, 2.56)	0.40	1.20(0.76, 1.89)	0.43	0.84(0.66, 1.08)	0.17
Paternal occupation-non-farmer	2.45(0.88, 6.86)	0.09	0.77(0.34, 1.72)	0.52	0.93(0.63, 1.37)	0.70
Household wealth per tertile increase^b^	1.17(0.69, 1.96)	0.56	0.83(0.61, 1.14)	0.24	0.95(0.80, 1.12)	0.54
***Randomized prenatal regimen by supplementing duration***						
Folic acid or folic acid plus iron <180 days	1.00 (Reference)		1.00 (Reference)		1.00 (Reference)	
Folic acid plus iron ≥180 days	0.55(0.18, 1.66)	0.29	1.20(0.66, 2.18)	0.56	1.02(0.73, 1.41)	0.94
Multiple micronutrients <180 days	0.71(0.25, 1.96)	0.50	1.30(0.74, 2.31)	0.36	0.94(0.68, 1.31)	0.73
Multiple micronutrients ≥180 days	0.14(0.02, 1.07)	0.06	0.37(0.16, 0.90)	0.03	0.63(0.44, 0.89)	0.01
Child sex-Female	0.49(0.21, 1.13)	0.09	0.81(0.51, 1.29)	0.37	0.81(0.63, 1.03)	0.08
Preterm (<37 gestation weeks)	2.62(0.69, 9.99)	0.16	2.15(0.87, 5.27)	0.11	0.73(0.37, 1.44)	0.37
Small for gestational age (<10^th^ percentile)	4.94(2.16, 11.33)	<0.001	1.85(0.98, 3.50)	0.06	1.63(1.13, 2.33)	0.01
Low birth weight (<2500 g)^c^	10.60(3.57, 31.49)	<0.001	2.50(0.87, 7.17)	0.09	2.18(1.11, 4.29)	0.02

^a^“Subgroup 4: Consistently above average” was the comparison group. Variables were included in the multinomial logistic regression analyses for their p values in the one-way analysis of variance or Chi-Square tests (Supplementary Table S3) less than 0.10.

^b^Maternal and paternal education were categorized as <3 years, Primary, Secondary and High school+, respectively. Household wealth was categorized as low, medium and high, respectively. All the variables were then treated as continuous variables in the multinomial logistic regression analyses.

^c^The results for low birth weight were adjusted for the variables in the main model but excluding preterm and small for gestational age. Abbreviations: RR, relative risk; CI, confidence interval.

We also examined these associations between Subgroup 1 and 2, and between Subgroup 3 and 4, respectively, and similar predictors were observed (Supplementary Table S4).

### Relationships of development trajectory group with middle childhood and adolescent development outcomes

As shown in Table 3, the children who performed consistently above average during the first 2 years of life had persistently higher test scores in middle childhood and early adolescence. Infants that were consistently above average during the first two years had 8.02 (95% CI 1.46, 14.59) and 2.52 (95% CI 0.62, 4.41) points higher cognitive test scores in adolescence as compared to those who started below average and then declined or those who were consistently average, respectively.

**Table 3:** Associations of identified cognitive development trajectories in the first two years of life with cognitive outcomes at 30 months of age, middle childhood and early adolescence.

		MDI at 30 months N = 1099	FSIQ at middle childhood N = 669	FSIQ at early adolescence N = 735
Subgroup 1: Start below average-then decrease	N	27	21	15
	Mean (SD)	55.1 (27.2)	76.8 (12.8)	88.0 (15.0)
Subgroup 2: Start below average-then increase	N	83	36	43
	Mean (SD)	81.5 (16.2)	91.3 (12.9)	97.0 (12.0)
Subgroup 3: Consistently average	N	454	256	294
	Mean (SD)	80.0 (17.5)	87.0 (11.4)	96.0 (12.0)
Subgroup 4: Consistently above average	N	535	356	383
	Mean (SD)	94.7 (15.0)	91.9 (12.7)	100.0 (12.0)
**Unadjusted mean differences (95% CI)**				
Subgroup 1 vs Subgroup 4 as reference		−39.59 (−50.15, −29.04)	−15.11 (−20.32, −9.91)	−12.01 (−18.08, −5.94)
Subgroup 2 vs Subgroup 4 as reference		−13.24 (−16.97, −9.51)	−0.62 (−4.55, 3.31)	−3.94 (−6.13, −1.75)
Subgroup 3 vs Subgroup 4 as reference		−14.67 (−16.71, −12.64)	−4.97 (−6.77, −3.18)	−4.72 (−6.69, −2.75)
Subgroup 2 vs Subgroup 3 as reference		1.43 (−2.58, 5.44)	4.36 (0.20, 8.51)	0.78 (−2.30, 3.87)
Subgroup 1 vs Subgroup 2 as reference		−26.35 (−37.47, −15.23)	−14.50 (−20.38, −8.61)	−8.07 (−14.56, −1.58)
**Adjusted mean differences (95% CI)**^**a**^				
Subgroup 1 vs Subgroup 4 as reference		−35.52 (−45.38, −25.66)	−10.20 (−14.41, −5.99)	−8.02 (−14.59, −1.46)
Subgroup 2 vs Subgroup 4 as reference		−10.93 (−14.80, −7.06)	1.75 (−2.97, 6.48)	−2.58 (−4.88, −0.28)
Subgroup 3 vs Subgroup 4 as reference		−12.76 (−14.86, −10.65)	−2.92 (−4.31, −1.52)	−2.52 (−4.41, −0.62)
Subgroup 2 vs Subgroup 3 as reference		2.34 (−1.52, 6.20)	3.59 (−1.20, 8.38)	0.61 (−2.67, 3.90)
Subgroup 1 vs Subgroup 2 as reference		−25.16 (−35.13, −15.19)	−11.43 (−19.69, −3.18)	−6.98 (−13.47, −0.49)

^a^Adjusted for assessors and potential confounding factors including parental age, job and education at pregnancy enrollment, household wealth at pregnancy enrollment, maternal MUAC at pregnancy enrollment, maternal parity, randomized regimen, birth outcome (SGA), and sex in general estimating equation linear models.

Abbreviations: CI, confidence interval; FSIQ, full-scale intelligence quotient derived from WISC-IV, Wechsler Intelligence Scale for Children-Fourth Edition; SD, standard deviation; SGA, small for gestational age; MUAC, mid upper arm circumference.

In the adjusted analyses (Table 3), infants in the trajectory group that started below average and then improved and the group that were consistently average did not differ in cognitive outcomes in middle childhood or adolescence. However, the cognitive deficits of trajectory group that started below average and then declined relative to group that started below average and then improved persisted into adolescence with an adjusted mean FISQ differences of −6.98 (95% CI −13.47, −0.49).

We conducted a sensitivity analysis using IPW to account for potential bias due to outcome censoring (loss to follow-up) and found there were no qualitative differences in our findings (Supplemental Table 5).

We also examined components of the FSIQ score and found similar associations within the VCI, WMI, PRI and PSI scores (Supplementary Tables S6 and Table S7).

### Relationships of single-time point BSID-II scores at 12 and 24 months with middle childhood and adolescent development outcomes

We observed statistically significant, but weaker correlations magnitude of association, between BSID-II tertiles and development outcomes in middle childhood and early adolescence (Supplementary Table S8). Young children in the highest tertile of development scores at 12 and 24 months had 2.57 (95% CI: 0.31, 4.82) and 4.67 (95% CI: 2.01, 7.33) points higher scores in early adolescence as compared to those in the lowest tertile, respectively.

## Discussion

We identified four distinct trajectories of infant cognitive development during the first two years of life in rural China: (1) children who started below average and then declined, (2) children who started below average and then improved, (3) children who were consistently average and (4) children who performed consistently above average. Higher maternal education and supplementing antenatal multiple micronutrients beyond 180 days were associated with reduced risk of being in suboptimal development trajectories; while, SGA and low birth weight birth increased the risk of being in the suboptimal groups. The developmental advantages of Subgroup 4 (consistently above average) over the other three trajectory groups persisted through middle childhood into early adolescence. In addition, the infants from subgroup 1 (started below average-then decreased) had the lowest test scores in middle childhood and early adolescence. These findings suggest that infant cognitive development trajectories are robust predictors of children long-term development outcomes.

We used a data-driven approach with five repeated measures in generally healthy children to identify development trajectories. One longitudinal study from Australia reported similar trajectories in aspect of language development between 8 and 48 months of age, which used the latent class analysis to address the categorized measures^13^. However, to the best of our knowledge, our study is the first to assess the relationship of early life development with later life outcomes with these methods. This method advancement shows that trajectory modeling can identify subgroups of children who share similar important longitudinal changes in development during the first two years of life. We also found the trajectory approach provided greater contrast between infants as compared to single time-point assessment of BSID-II scores. These subgroups are also not distinct in studies that report development trajectory as age-specific averages^11,12^. As a result, trajectory modeling may better capture the dynamic process of child development and thus provide stronger associations with long-term outcomes.

We identified a pronounced catch-up pattern in Subgroup 2 (started below average-then increased) that would have been missed with a single time point assessment. In this study, the catch-up development appeared to start early in infancy and provides additional evidence that the first two years of life are critical for development. Of note, the catch-up pattern of cognitive development was similar to that of physical growth which also generally starts early in the first months of life^14,15^. In addition, our risk factor analyses suggest that a combination of prenatal nutrition, socioeconomic, and environmental factors may affect catch-up development. Although this finding requires replication in other studies, some studies reported that improvements in exclusive breastfeeding and longer partial breastfeeding were associated with better cognitive development outcomes later in life^16,17^. In addition, the identification of Subgroup 1 (started below and then declined) indicates that the trajectory modeling can be used to identify children with high risk of occurring delayed development. As a result, assessment of cognitive development trends may provide valuable information on children’s development and long-term outcomes.

Our study was also able to explore the early determinants that underlie the heterogeneity in these trajectories, which may help develop intervention strategies to reduce the risk of high-risk children being in suboptimal trajectories. We found that infants from high-income household wealth were more likely to be in the above average group, which is similar to a study conducted in the UK among subjects aged 2–16^18^. In addition, our finding that maternal education could independently act as a beneficial factor for child optimal development was in agreement with previous studies^19–21^. It has been proposed that higher maternal education level was associated with less maternal depression, better child nutrition status, child-rearing environment and ability to access and benefit from interventions. As described by the early nutrition programming^22^, antenatal micronutrient supplementation may improve child development, but the evidence has been inconsistent in the studies that assessed outcome at a single point^23–25^. In the present study, infants born by women who consumed multiple micronutrients for 180 days or beyond during pregnancy were less likely to be in the suboptimal trajectories. This finding extends the current knowledge on mechanisms underlying the antenatal micronutrient supplementation and long-term outcomes, which may have been linked during early life but would not be captured using the single-time assessment.

We also identified SGA or LBW as another predictor of development trajectories. These findings are consistent with prior studies^26,27^. Although some studies with short periods of follow-up reported that the influence of intrauterine growth restriction on cognitive development appeared to diminish overt time^28^, our finding and another study from UK and Ireland supported that impaired cognitive trajectories set in early life might persist into adolescence and early adulthood^11^. One recent systematic review from South Asia also reported that LBW children (<10 ys) relative to normal children had 5 points lower cognitive scores with a dose-response relationship^29^. Further, the differences among them became bigger along with the increasing age^29^, which was similar to the tendency observed in Subgroup 1 trajectory, i.e., linearly declining after birth. In the present study, this Subgroup 1 trajectory (started below average and then declined) was characterized by high risk of occurring preterm, LBW, and/or SGA (Supplementary Table S3). Hence, these findings highlight the programs that aim to reduce the risk of adverse birth outcomes before and during pregnancy.

There remains debate as to the long-term functional outcomes of cognitive tests in infancy in LMIC settings^30^. After using structural equation model to account for measurement error with 3 repeated measures among 130 infants, one longitudinal investigation from US reported that infant cognitive function moderately correlated with adolescent development outcomes with a correlation coefficient of 0.57^31^, which was higher than that observed in our study (0.18 for 12 months and 0.30 for 24 months, respectively; Supplementary Table S8). Taken together, these findings suggest that using single-time assessment of infant cognitive development has limitations to predict long-term outcomes and identify high-risk children for delayed development. In the present study, adolescents from Subgroup 4 (consistently above average) had the highest cognitive test scores, suggesting that the developmental advantages established in early life could persist through middle childhood into early adolescence. In addition, the infants from subgroup 1 (started below average-then decreased) had the lowest test scores in middle childhood and early adolescence. These findings suggest that infant cognitive development trajectories are strong predictors of children long-term development outcomes, and highlight the importance of providing appropriate interventions as early as possible, which could ameliorate restricted development and have important implications for human capital and well-being across the life course.

The results in the present study should be interpreted with a few limitations. First, the follow-up rate for children in middle childhood and adolescence in the present study was approximately 50% of the original cohort and therefore bias due to dependent censoring is possible. Nevertheless, we found minimal to no differences in background characteristics for children who had development assessed as compared to those who did not. Besides, a sensitivity analysis using IPW to account for outcome censoring also suggested the main study findings were robust and the risk of bias due to censoring was likely minimal. Second, the use of data-driven approach allowed us to identify distinct cognitive trajectories over age and was appropriate for several repeated measurements of the same individuals. However, the trajectory modelling approach shares inherent limitations including that extracting the optimal number of subgroups, which is a process guided by statistical fit indices and some degree of investigators’ decision, and that the size of each trajectory was produced by the model that may result in small sizes and consequently limited power to further analysis. Third, the cognitive development trajectories that we identified in our study population in rural China may not be directly applicable to other settings. Finally, the underlying biological mechanisms between these predictors and cognitive trajectories cannot be examined in the present study.

In summary, we identified groups of distinct trajectories of cognitive development during the first two years of life in rural China. Prospectively, we found that these trajectory groups robustly predicted development scores through middle childhood into adolescence. In addition, our risk factor analyses indicated that integrated of nutritional, environmental, and educational interventions during the first 1,000 days of life may affect early life cognitive development trajectories and produce long-term effects on development and human capital across the life course.

## Methods

### Participants

We used data from a prospective birth cohort of children born to women who participated in a randomized, double-blind trial of antenatal micronutrient supplementation in rural western China. Children were followed in early childhood (age 3 to 30 months), middle childhood (age 7–9 years) and early adolescence (age 10–12 years). Details and procedures of the trial and follow-up studies have been described elsewhere^23–25,32^.

Briefly, all pregnant women across villages from two counties were randomized to take a daily capsule of either folic acid, folic acid plus iron, or multiple micronutrients between August 2002 and February 2006. In the trial 4604 singleton births occurred, and 1400 births born in 2004–2006 were enrolled in long-term follow-up cohort. A total of 1388 was enrolled after excluding deaths (n = 3), birth defects (n = 7) and disabled parents (n = 2). Among them, 660 were followed at 7–9 years of age between October 2012 and September 2013, and 735 at 10–12 years of age between June 2016 and December 2016 for cognitive assessment.

### Assessments of cognitive development

At the 3, 6, 12, 18, 24- and 30-month visit, mental development (MD) was assessed using a culturally appropriate, and locally validated Chinese version of Bayley Scales of Infant Development (BSID-II)^33^. MD raw scores were transformed into age-standardized scores based on the data for infants in US^34^.

In middle childhood and adolescence, we used the Wechsler Intelligence Scale for Children, Fourth Edition (WISC-IV) to assess cognitive development^35^. According to Chinese norms of WISC-IV with satisfied reliability and validity, age-standardized full-scale intelligence quotient (FSIQ), representing the general cognitive development, and aspects of verbal comprehension (VCI), perceptual reasoning (PRI), working memory (WMI), and processing speed index (PSI) were derived^36^.

Cognitive tests were standardly administered by public health graduates at subjects’ own home, local school or hospital meeting room that were free of distractions. Field staff administering these cognitive tests were unaware of the socioeconomic background, randomized treatment allocation, birth outcomes or other health status of participants.

### Covariates

Information on socioeconomic status (parental age, occupation, education and household wealth), maternal nutrition status before pregnancy (mid-upper arm circumference), randomized regimen (folic acid, folic acid plus iron, and multiple micronutrients), maternal parity and birth outcomes (preterm birth, low birth weight [LBW], small for gestational age [SGA] birth and infant sex) was collected as part of the original trial using standard questionnaire, methods and/or procedures. These details are documented elsewhere^23^. A wealth index was established from an inventory of 16 household assets or facilities by principal component analysis, which was then classified into thirds as an indicator of low-, middle- and high-income households^37^. Preterm birth was defined as babies born alive before 37 weeks’ gestational age, and low birth weight was defined as a birth weight of less than 2500 g, as per the World Health Organization (WHO) guidelines^38,39^. According to Intergrowth standards, SGA birth was defined as birth weight below the 10th percentile of weight-for-age and sex^40^.

Given our prior findings that multiple micronutrient supplementation could significantly improve cognitive development with the largest benefits observed with supplementation of at least 180 days^25^, we combined the randomized treatment regimens and duration into a categorical variable, i.e., folic acid or iron/folic acid lasting for <180 days (as reference), iron/folic acid lasting for ≥180 days, multiple micronutrients lasting for <180 days, and multiple micronutrients lasting for ≥180 days.

### Statistical analysis

To increase comparability across ages, we transformed the age-standardized cognitive test scores into z-scores based on age-specific medians and SD within the sample. We then used a group‐based trajectory modelling, specifically the “traj” macro in Stata, to identify infant cognitive z-score developmental trajectories across 3, 6, 12, 18, and 24 months of age^41–43^. GBTM can identify subgroups of individuals who share similar patterns of development^42,43^, and has been used to identify distinct trajectories of body composition and body mass index (BMI) that are associated with risks for obesity, asthma, morbidity and mortality later in life^44–51^. Models with two or more subgroups were compared to identify the optimal number of subgroups and shapes that best characterized the data, with maximum likelihood estimation accounting for missing z scores at any time point. The final model was selected based on general recommendations including: (i) tests for parameter estimates for linear, quadratic and cubic terms, (ii) Bayesian and Akaike information criterion value, (ii) average of the posterior probabilities of group membership for individuals assigned to each group, (iv) odds of correct classification based on the posterior probabilities of group membership, and (v) minimizing overlap in confidence intervals (CIs) while summarizing the distinctive features of the data as parsimonious as possible^43^.

Once the most appropriate trajectories were derived, the subgroup categories variable was used in all subsequent analyses. Baseline characteristics by the trajectory groups were compared using Chi-squared tests or analysis of variance, and multivariate multinomial logistic regression models. We then used generalized estimating equations with an independent correlation structure to assess the relationship of the trajectory groups with cognitive development outcomes at 30 months, middle childhood and early adolescence. To hand the missing data of cognitive outcome in middle childhood and early adolescence and examine its potential to influence the results, we applied the inverse probability weighting (IPW)^52^. We also conducted analyses using single-time point Bayley scores at 12 and 24 months to compare the magnitude of association for the GBTM approach.

Age-standardized FSIQ and VCI, WMI, PRI, and PSI scores were taken as the primary and secondary outcomes, respectively. All statistical analyses were performed using Stata 12.0 (Stata Corp, College Station, Texas, USA).

### Ethical approval

The protocols of the original trial and all follow-up studies conformed to the ethical principles of the 1964 Declaration of Helsinki, and were approved by UNICEF and the ethics committee of Xi’an Jiaotong University Health Science Center. Informed written content from pregnant women, and parents/caregivers, and oral consent from children were obtained.

## Supplementary information

Supplementary Online Content

## Data Availability

All data generated or analysed during this study are included in this published article (and its Supplementary Information files).
