# Supplementary material for: Early life cognitive development trajectories and intelligence quotient in middle childhood and early adolescence in rural western China
Source: Sci Rep. 2019 Dec 4;9:18315. doi: 10.1038/s41598-019-54755-1 (PMC6892923; doi:10.1038/s41598-019-54755-1)
Supplement: Supplementary file 1 — Supplementary Online Content [file 41598_2019_54755_MOESM1_ESM.pdf]

Supplementary Online Content

Early life cognitive development trajectories and intelligence quotient in middle childhood and early adolescence in rural western China

Zhonghai Zhu, Suying Chang, Yue Cheng, Qi Qi, Shaoru Li, Mohamed Elhoumed, Hong Yan, Michael J Dibley, Wafaie W. Fawzi, Lingxia Zeng, Christopher R. Sudfeld

Supplementary Table S1. Comparisons of background characteristics between individuals lost to follow-up and those followed at middle childhood and adolescence

Supplementary Table S2 Fit indices for identified trajectories for cognitive development during the first two years of life

Supplementary Table S3. Characteristics of cognitive development trajectories during the first two years of life in a Chinese birth cohort study

Supplementary Table S4. Predictors of cognitive developmental trajectories identified in early childhood from a Chinese birth cohort study

Supplementary Table S5. Associations of identified cognitive development trajectories in the first two years of life with cognitive outcomes in middle childhood and early adolescence: using inverse probability weighting to account for missing outcome data

Supplementary Table S6. Associations of identified cognitive development trajectories in the first two years of life with WISC-IV components (VCI, WMI, PRI, PSI) at middle childhood

Supplementary Table S7. Associations of identified cognitive development trajectories in the first two years of life with WISC-IV components (VCI, WMI, PRI, PSI) at early adolescence

Supplementary Table S8. Tertiles of mental development score at the 12 and 24 months and cognitive outcomes at middle childhood and early adolescence

Supplementary Fig. S1. Flow chart of study participants

Supplementary Table S1 Comparisons of background characteristics between individuals lost to follow-up and those followed at middle childhood and adolescence

Factors	Middle childhood/ No. (%)/Mean (SD)			Adolescence/ No. (%)/Mean (SD)		
	Completed assessments	Loss to follow-up	P values	Completed assessments	Loss to follow-up	P values
N	669(48.2)	719(51.8)		735(53.0)	653(47.1)	
Maternal age	24.7(4.5)	24.1(4.4)	0.02	24.4(4.5)	24.3(4.4)	0.52
Maternal education			0.08			0.02
< 3 years	35(5.2)	26(3.6)		30(4.1)	31(4.8)	
Primary	174(26.1)	167(23.3)		189(25.8)	152(23.3)	
Secondary	383(57.3)	415(57.9)		399(54.4)	399(61.2)	
High school+	76(11.4)	109(15.2)		115(15.7)	70(10.7)	
Maternal occupation			0.07			0.32
Farmer	576(86.6)	596(83.1)		615(83.9)	557(85.8)	
Others	89(13.4)	121(16.9)		118(16.1)	92(14.2)	
Paternal age (years)	28.0(4.4)	27.4(4.1)	0.01	27.7(4.2)	27.7(4.3)	0.91
Paternal education			0.70			0.09
< 3 years	7(1.1)	5(0.7)		9(1.2)	3(0.5)	
Primary	68(10.2)	68(10.2)		80(10.9)	59(9.1)	
Secondary	446(67.0)	446(67.0)		465(63.5)	449(69.1)	
High school+	145(21.8)	145(21.8)		178(24.3)	139(21.4)	
Paternal occupation			<0.001			0.29
Farmer	536(80.2)	518(72.1)		549(74.9)	505(77.3)	
Others	132(19.8)	200(27.9)		184(25.1)	148(22.7)	

Factors	Middle childhood/ No. (%)/Mean (SD)			Adolescence/ No. (%)/Mean (SD)		
	**Completed assessments**	**Loss to follow-up**	**P values**	**Completed assessments**	**Loss to follow-up**	**P values**
Household wealth at enrollment			0.20			0.61
Low	170(25.4)	154(21.4)		179(24.4)	145(22.2)	
Medium	266(39.8)	296(41.2)		291939.6）	271(41.5)	
High	233(34.8)	269(37.4)		265(36.1)	237(36.3)	
Parity at enrollment			0.07			0.94
0	439(65.6)	504(70.1)		500(68.0)	443(67.8)	
≥1	230(34.4)	215(29.9)		235(62.0)	210(32.2)	
Maternal MUAC (cm)			0.56			0.08
<21.5	110(16.5)	109(15.3)		104(14.3)	115(17.7)	
≥21.5	557(83.5)	602(84.7)		624(85.7)	535(82.3)	
Trial regimen			0.18			0.32
Folic acid	246(36.8)	250(34.8)		265(36.1)	231(35.4)	
Iron/folic acid	209(31.2)	258(35.9)		235(32.0)	232(35.5)	
Multiple micronutrients	214(32.0)	211(29.4)		235(32.0)	190(29.1)	
Child characteristics						
Male	404(60.4)	441(61.3)	0.72	448(61.0)	397(60.8)	0.95
Birth weight (g)	3170(420)	3201(399)	0.16	3197(401)	3173(419)	0.28
Gestation weeks at birth	39.9(1.6)	39.9(1.5)	0.85	39.9(1.5)	39.9(1.6)	0.97
Preterm (<37 weeks gestation)	27(4.0)	23(3.2)	0.40	19(2.6)	31(4.8)	0.03
Low birth weight (<2500g)	36(5.5)	14(2.0)	0.001	25(3.4)	25(3.9)	0.65
Small for gestational age ($<10^{th}$ percentile)	87(13.4)	78(11.3)	0.24	82(11.5)	83(13.3)	0.33

Supplementary Table S2 Fit indices for identified trajectories for cognitive development during the first two years of life

Number of groups	Trajectory shapes[a]	P values for the test of parameters	BIC[b]	AIC[b]	APP[c]	OCC[d]	Size (%)
2	1 1	0.22/0.54	-6621.68	-6606.21	0.82/0.90	85/41	25.52/74.48
3	1 1 1	0.01/0.63/0.90	-6574.95	-6551.75	0.84/0.82/0.80	941/25/48	2.94/58.54/38.52
4	1 1 1 1	0.001/<0.001/0.004/0.13	-6569.13	-6538.18	0.81/0.66/0.67/0.76	668/94/27/20	3.14/12.28/33.57/51.00
5	1 1 1 1 1	0.001/0.01/0.002/0.04/0.007	-6565.80	-6527.12	0.79/0.68/0.68/0.64/0.65	559/124/29/12/112	3.26/10.54/31.15/42.20/12.84
4	2 2 2 2	0.15/0.03/0.79/0.02	-6575.98	-6534.73	0.83/0.67/0.67/0.77	882/103/27/20	2.99/11.89/33.59/51.53
4	3 3 3 3	0.41/0.001/0.001/0.68	-6579.16	-6527.59	0.83/0.71/0.70/0.77	918/132/29/21	2.93/10.79/35.86/50.42
4	1 3 3 1	<0.001/<0.001/0.001/0.05	-6568.19	-6526.94	0.81/0.72/0.71/0.75	738/152/25/21	3.18/10.24/40.26/46.32
4	1 3 3 2	0.001/0.41/0.96/0.93	-6572.72	-6528.88	0.84/0.72/0.79/0.76	1087/189/53/17	2.69/10.70/31.42/35.18

[a]Trajectory shapes: 1=linear, 2=quadratic, 3=cubic.

[b]BIC, Bayesian information criterion. AIC, Akaike information criterion. The model with the highest (least negative) value of BIC and AIC is preferred.

[c]APP, an average of the posterior probabilities of group membership for individuals assigned to each group, should exceed 0.7, range 0.00-1.00.

[d]OCC, odds of correct classification based on the posterior probabilities of group membership, should exceed 5.

Supplementary Table S3 Characteristics of cognitive development trajectories during the first two years of life in a Chinese birth cohort study

Items	Mean (SD)/ n (%)				P values for differences among groups
	Subgroup 1: Start below average-then decrease (N=35)	Subgroup 2: Start below average-then increase (N=99)	Subgroup 3: Consistently average (N=530)	Subgroup 4: Consistently above average (N=724)	
Socioeconomic characteristics at enrollment[a]					
Maternal age	24.6(3.6)	24.1(4.5)	24.5(4.6)	24.3(4.4)	0.81
Maternal education					<0.001
< 3 years	5(14.3)	10(10.1)	27(5.1)	19(2.6)	
Primary	18(51.4)	31(31.3)	151(28.6)	141(19.5)	
Secondary	11(31.4)	49(49.5)	303(57.4)	435(60.2)	
High school+	1(2.9)	9(9.1)	47(8.9)	12(17.7)	
Maternal occupation					<0.001
Farmer	34(97.1)	91(91.9）	471(89.4)	576(79.9)	
Others	1(2.9)	8(8.1)	56(10.6)	145(20.1)	
Paternal age (years)	28.1(3.8)	27.3(4.2)	27.9(4.4)	27.6(4.1)	0.38
Paternal education					<0.001
< 3 years	1(2.9)	1(1.0)	8(1.5)	2(0.3)	
Primary	5(14.3)	14(14.1)	63(11.9)	57(7.9)	
Secondary	22(62.9)	64(64.7)	366(69.2)	462(64.3)	
High school+	7(20.0)	20(20.2)	92(17.4)	198(27.5)	
Paternal occupation					<0.001

Items	Mean (SD)/ n (%)				P values for differences among groups
	**Subgroup 1: Start below average-then decrease (N=35)**	**Subgroup 2: Start below average-then increase (N=99)**	**Subgroup 3: Consistently average (N=530)**	**Subgroup 4: Consistently above average (N=724)**	
Farmer	28(80.0)	85(85.9)	430(81.1)	511(70.8)	
Others	7(20.0)	14(14.1)	100(18.9)	211(29.2)	
Household wealth					0.001
Low	11(31.4)	33(33.3)	131(24.7)	149(20.6)	
Medium	12(34.3)	39(39.4)	235(44.3)	276(38.1)	
High	12(34.3)	27(27.3)	164(30.9)	299(41.3)	
Pregnancy					
Parity at enrollment					0.22
0	22(62.9)	69(69.7)	344(64.9)	508(70.2)	
≥1	13(37.1)	30(30.3）	186(35.1)	216(29.8)	
MUAC (cm)	22.6(1.4）	23.3(1.9）	23.2(1.8)	23.2(1.8)	0.26
<21.5	8(22.3)	15(15.2)	86(16.4)	110(15.3)	0.67
≥21.5	27(77.1)	84(84.9)	440(83.7)	608(84.7)	
Randomized prenatal regimen					0.11
Folic acid	18(51.1)	33(33.3)	188(35.5)	257(35.5)	
Iron/folic acid	11(31.4)	38(38.4)	192(36.2)	226(31.2)	
Multiple micronutrients	6(17.1)	28(28.3)	150(28.3)	241(33.3)	
Randomized prenatal regimen by duration					0.02
Folic acid or folic acid/ iron <180 days	25(71.4)	53(53.5)	289(54.5)	369(51.0)	

Items	Mean (SD)/ n (%)				P values for differences among groups
	Subgroup 1: Start below average-then decrease (N=35)	Subgroup 2: Start below average-then increase (N=99)	Subgroup 3: Consistently average (N=530)	Subgroup 4: Consistently above average (N=724)	
folic acid/iron ≥180 days	4(11.4)	18(18.2)	91(17.2)	114(15.8)	
Multiple micronutrient <180 days	5(14.3)	21(21.1)	87(16.4)	116(16.0)	
Multiple micronutrient ≥180 days	1(2.9)	7(7.1)	63(11.9)	125(17.3)	
Birth outcomes					
Child sex					
Male	27(77.1)	65(65.7)	338(63.8)	415(57.3)	0.01
Female	8(22.9)	34(34.3)	192(36.2)	309(42.7)	
Birth weight	2915(464)	3107(383)	3176(430)	3216(389)	<0.001
Preterm (<37 gestation w eeks)	3(8.6)	7(7.1)	141(2.6)	26(3.6)	0.06
Small for gestational age (<10th percentile)	9(26.5)	13(14.3)	75(14.6)	68(9.7)	0.004
Low birth weight (<2500g)	6(17.7)	5(5.3)	24(4.6)	15(2.1)	<0.001

Abbreviation: MUAC, mid-upper arm circumference; SD, standard deviation.

[a]Socioeconomic factors were assessed at time of enrollment in original trial.

Supplementary Table S4 Predictors of cognitive developmental trajectories identified in early childhood from a Chinese birth cohort study[a]

Factors	Start below average-then decrease vs. Start below average-then increase		Consistently average vs. Consistently above average	
	RR (95% CI)	P values	RR (95% CI)	P values
Maternal education per grade increase[b]	0.57(0.40, 0.83)	0.003	0.87(0.78, 0.97)	0.01
Maternal occupation-non-farmer	0.41(0.06, 2.99)	0.38	0.79(0.56, 1.12)	0.19
Paternal education per grade increase[b]	1.09(0.74, 1.61)	0.66	0.91(0.80, 1.03)	0.15
Paternal occupation-non-farmer	1.94(1.02, 3.70)	0.04	0.96(0.76, 1.21)	0.74
Household wealth per tertiles increase[b]	1.28(0.87, 1.89)	0.22	0.96(0.88, 1.06)	0.44
Randomized prenatal regimen by supplementing durations				
Folic acid or folic acid plus iron <180 days	1.00 (Reference)		1.00 (Reference)	
folic acid plus iron ≥180 days	0.65(0.23, 1.82)	0.41	1.01(0.85, 1.19)	0.92
Multiple micronutrient <180 days	0.69(0.31, 1.55)	0.37	0.97(0.80, 1.18)	0.76
Multiple micronutrient ≥180 days	0.53(0.07, 3.82)	0.53	0.76(0.61, 0.95)	0.02
Child sex-Female	0.6(0.35, 1.34)	0.27	0.89(0.77, 1.01)	0.07
Preterm (<37 gestation weeks)	1.23(0.44, 3.41)	0.69	0.82(0.52, 1.30)	0.40
Small for gestational age (<10th percent ile)	1.74(0.98, 3.10)	0.06	1.27(1.08, 1.49)	0.003
Low birth weight (<2500g)[c]	2.19(1.17, 4.11)	0.01	1.42(1.08, 1.85)	0.01

[a]The trajectories were characterized as “Subgroup 1: Start below average-then decrease”, “Subgroup 2: Start below average-then increase”, “Subgroup 3: Consistently average”, and “Subgroup 4:

Consistently above average", respectively. Variables were included for their p values in the one-way analysis of variance or Chi-Square tests (table 3) less than 0.10 in the generalized estimating equations models with log-link, assuming Poisson distribution. And, the analyses were performed between subgroup 1 and 2, and between subgroup 3 and 4, with Subgroup 2 and 4 as reference subgroup, respectively.

[b]Maternal and paternal education were categorized as < 3 years, Primary, Secondary and High school+, respectively. Household wealth was categorized as low, medium and high, respectively. All the variables were then treated as continuous variables in the regression analyses.

[c]The results for low birth weight were adjusted for the variables in the main model but excluding preterm and small for gestational age.

Supplementary Table S5 Associations of identified cognitive development trajectories in the first two years of life with cognitive outcomes in middle childhood and early adolescence: using inverse probability weighting to account for missing outcome data

		FSIQ at middle childhood N=669	FSIQ at early adolescence N=735
Subgroup 1: Start below average-then decrease	N	21	15
	Mean (SD)	76.8(12.8)	88.0(15.0）
Subgroup 2: Start below average-then increase	N	36	43
	Mean (SD)	91.3(12.9)	97.0(12.0)
Subgroup 3: Consistently average	N	256	294
	Mean (SD)	87.0(11.4)	96.0(12.0)
Subgroup 4: Consistently above average	N	356	383
	Mean (SD)	91.9(12.7)	100.0(12.0)
Unadjusted mean differences (95% CI)			
Subgroup 1 vs Subgroup 4 as reference		-15.20(-20.46, -9.94)	-12.09(-18.07, -6.10)
Subgroup 2 vs Subgroup 4 as reference		-0.70(-4.62, 3.23)	-3.95(-6.15, -1.76)
Subgroup 3 vs Subgroup 4 as reference		-5.01(-6.83, -3.19)	-4.74(-6.69, -2.78)
Subgroup 2 vs Subgroup 3 as reference		4.31(0.16, 8.46)	0.78(-2.30, 3.87)
Subgroup 1 vs Subgroup 2 as reference		-14.50(-20.41, -8.59)	-8.13(-14.55, -1.71)
Adjusted mean differences (95% CI)[a]			
Subgroup 1 vs Subgroup 4 as reference		-10.22(-14.44, -6.00)	-8.13(-14.62, -1.64)
Subgroup 2 vs Subgroup 4 as reference		1.72(-3.02, 6.46)	-2.60(-4.89, -0.31)
Subgroup 3 vs Subgroup 4 as reference		-2.93(-4.32, -1.53)	-2.54(-4.44, -0.65)
Subgroup 2 vs Subgroup 3 as reference		3.57(-1.23, 8.37)	0.62(-2.62, 3.86)
Subgroup 1 vs Subgroup 2 as reference		-11.46(-19.71, -3.21)	-6.85(-13.35, -0.35)

[a]Adjusted for assessors and potential confounding factors including parental age, job and education at pregnancy enrollment, household wealth at pregnancy enrollment, maternal MUAC at pregnancy enrollment, maternal parity, randomized regimen, birth outcome (SGA), and sex in general estimating equation linear models.

Abbreviations: CI, confidence interval; FSIQ, full-scale intelligence quotient derived from WISC-IV, Wechsler Intelligence Scale for Children-Fourth Edition; SD, standard deviation; SGA, small for gestational age; MUAC, mid upper arm circumference.

Supplementary Table S6 Associations of identified cognitive development trajectories in the first two years of life with WISC-IV components (VCI, WMI, PRI, PSI) at middle childhood

		WISC-IV at middle childhood (N=669)			
		VCI	WMI	PRI	PSI
Subgroup 1: Start below average-then decrease	N	21	21	21	21
	Mean (SD)	75.8(11.2)	79.7(92.6)	84.6(15.0)	82.3(14.5)
Subgroup 2: Start below average-then increase	N	36	36	36	36
	Mean (SD)	90.9(16.0)	92.6(10.6)	95.1(15.0)	93.6(11.9)
Subgroup 3: Consistently average	N	256	256	256	256
	Mean (SD)	85.1(14.8)	89.3(10.5)	91.5(12.8)	93.5(11.6)
Subgroup 4: Consistently above average	N	356	356	356	356
	Mean (SD)	89.4(15.6)	93.2(12.6)	95.5(12.9)	97.6(13.1)
Unadjusted mean differences (95% CI)	1 vs 4 (ref.)	-13.51(-17.17, -9.89)	-13.46(-20.08, -6.84)	-10.90(-16.22, -5.59)	-15.27(-21.43, -9.11)
	2 vs 4 (ref.)	1.63(-3.41,6.67)	-0.57(-4.05,2.91)	-0.44(-6.08,5.20)	-3.95(-6.01, -1.89)
	3 vs 4 (ref.)	-4.25(-6.51, -2.00)	-3.93(-5.52, -2.33)	-4.04(-5.79, -2.29)	-4.04(-5.94, -2.14)
	2 vs 3 (ref.)	5.88(0.88, 10.89)	3.36(-0.42, 7.15)	3.60(-1.87, 9.07)	0.10(-2.57, 2.76)
	1 vs 2 (ref.)	-15.13(-20.35, -9.92)	-12.90(-19.54, -6.26)	-10.46(-18.13, -2.80)	-11.33(-17.76, -4.89)
Adjusted mean differences (95% CI)[a]	1 vs 4 (ref.)	-9.16(-11.46, -6.85)	-9.22(-14.38, -4.06)	-8.01(-13.53, -2.50)	-10.88(-16.03, -5.73)
	2 vs 4 (ref.)	3.60(-0.61, 7.80)	2.16(-1.79, 6.11)	0.21(-6.26, 6.68)	-1.37(-4.35,1.61)
	3 vs 4 (ref.)	-2.20(0.38, 4.02)	-2.41(-3.55, -1.26)	-2.74(-4.74, -0.74)	-2.34 (-3.91, -0.77)
	2 vs 3 (ref.)	4.52(-0.15, 9.19)	3.43(-0.32, 7.18)	3.10(-3.12, 9.31)	-0.17(-3.68, 3.34)
	1 vs 2 (ref.)	-10.77(-17.33, -4.22)	-10.99(-16.34, -5.63)	-10.04(-22.12, 2.03)	-7.70(-15.87, 0.47)

[a]Adjusted for assessors and potential confounding factors including parental age, job and education at pregnancy enrollment, household wealth at pregnancy enrollment, maternal middle-upper arm circumferences at pregnancy enrollment, parity, maternal pregnancy micronutrient supplements, birth outcome (small for gestational age), and sex in general estimating equation linear models.

Abbreviations: CI, confidence interval; FSIQ, full-scale intelligence quotient derived from WISC-IV, Wechsler Intelligence Scale for Children-Fourth Edition; SD, standard deviation.

Supplementary Table S7 Associations of identified cognitive development trajectories in the first two years of life with WISC-IV components (VCI, WMI, PRI, PSI) at early adolescence

		WISC-IV at early adolescence (n=735)			
		VCI	WMI	PRI	PSI
Subgroup 1: Start below average-then decrease	N	15	15	15	15
	Mean (SD)	92.0(15.0)	88.0(11.0)	90.0(21.0)	91.0(12.0)
Subgroup 2: Start below average-then increase	N	43	43	43	43
	Mean (SD)	103.0(18.0)	92.0(9.0)	96.0(12.0)	97.0(14.0)
Subgroup 3: Consistently average	N	294	294	294	294
	Mean (SD)	101.0(16.0)	93.0(12.0)	94.0(12.0)	98.0(13.0)
Subgroup 4: Consistently above average	N	383	383	383	383
	Mean (SD)	105.0(15.0)	96.0(11.0)	98.0(13.0)	102.0(13.0)
Unadjusted mean differences (95% CI)	1 vs 4 (ref.)	-12.97(-18.71, -7.22)	-7.67(-12.40, -2.93)	-8.03(-17.94, 1.87)	-11.54(-16.63, -6.45)
	2 vs 4 (ref.)	-1.63(-6.20, 2.95)	-4.33(-6.70, -1.96)	-1.68(-4.86, 1.50)	-5.80(-9.46, -2.14)
	3 vs 4 (ref.)	-3.85(-6.12, -1.58)	-2.60(-4.40, -0.80)	-3.50(-5.33, -1.68)	-4.61(-6.24, -2.97)
	2 vs 3 (ref.)	2.22(-2.93, 7.38)	-1.73(-4.70, 1.25)	1.82(-1.72, 5.36)	-1.19(-4.97, 2.58)
	1 vs 2 (ref.)	-11.34(-18.44, -4.24)	-3.34(-8.01, 1.34)	-6.35(-16.92, 4.21)	-5.74(-12.97, 1.49)
Adjusted mean differences (95% CI)[a]	1 vs 4 (ref.)	-9.67(-15.17, -4.16)	-5.29(-10.36, -0.22)	-5.93(-15.79, 3.93)	-7.21(-13.58, -0.84)
	2 vs 4 (ref.)	-1.41(-6.02, 3.19)	-3.19(-5.72, -0.66)	-0.64(-3.53, 2.25)	-3.76(-6.33, -1.19)
	3 vs 4 (ref.)	-1.40(-3.62, 0.81)	-1.27(-2.95, 0.40)	-2.12(-4.00, -0.24)	-3.20(-4.75, -1.64)
	2 vs 3 (ref.)	0.41(-4.97, 5.79)	-1.44(-4.67, 1.78)	2.38(-0.70, 5.44)	-0.29(-3.22, 2.65)
	1 vs 2 (ref.)	-8.78(-17.93, 0.37)	-1.02(-5.67, 3.62)	-8.00(-18.66, 2.66)	-5.41(-9.57, -1.26)

[a]Adjusted for assessors and potential confounding factors including parental age, job and education at pregnancy enrollment, household wealth at pregnancy enrollment, maternal middle-upper arm circumferences at pregnancy enrollment, parity, maternal pregnancy micronutrient supplements, birth outcome (small for gestational age), and sex in general estimating equation linear models.

Abbreviations: CI, confidence interval; FSIQ, full-scale intelligence quotient derived from WISC-IV, Wechsler Intelligence Scale for Children-Fourth Edition; SD, standard deviation.

Supplementary Table S8 Tertiles of mental development score at the 12 and 24 months and cognitive outcomes at middle childhood and early adolescence

Mental development	FSIQ at middle childhood				FSIQ at early adolescence			
	N	Mean (SD)	Adjusted mean differences (95% CI)[a]	Correlation coefficients (p values)	N	Mean (SD)	Adjusted mean differences (95% CI)[a]	Correlation coefficients (p values)
12 months				0.182 (<0.001)				0.175(<0.001)
Q1 (lowest)	205	86.6(12.4)	Reference		219	96.0(13.0)	Reference	
Q2	206	90.3(12.3)	2.74(1.91, 3.57)		221	99.0(13.0)	1.28(-0.96, 3.51)	
Q3 (highest)	173	92.0(12.0)	3.98(2.05, 5.92)		198	100.0(11.0)	2.57(0.31, 4.82)	
24 months				0.329 (<0.001)				0.295(<0.001)
Q1 (lowest)	203	85.7(11.1)	Reference		218	95.0(12.0)	Reference	
Q2	199	89.8(12.3)	3.51(1.45, 5.56)		210	98.0(13.0)	2.36(-0.51, 5.23)	
Q3 (highest)	188	94.1(12.4)	6.21(4.08, 8.34)		203	103(11.0)	4.67(2.01, 7.33)	

[a]Adjusted for assessors and potential confounding factors including parental age, job and education at pregnancy enrollment, household wealth at pregnancy enrollment, maternal MUAC at pregnancy enrollment, parity, randomized maternal pregnancy micronutrient supplements, birth outcome (SGA), and sex in general estimating equation linear models.

Abbreviations: CI, confidence interval; FSIQ, full-scale intelligence quotient derived from WISC-IV, Wechsler Intelligence Scale for Children-Fourth Edition; SD, standard deviation; SGA, small for gestational age; MUAC, mid upper arm circumference.

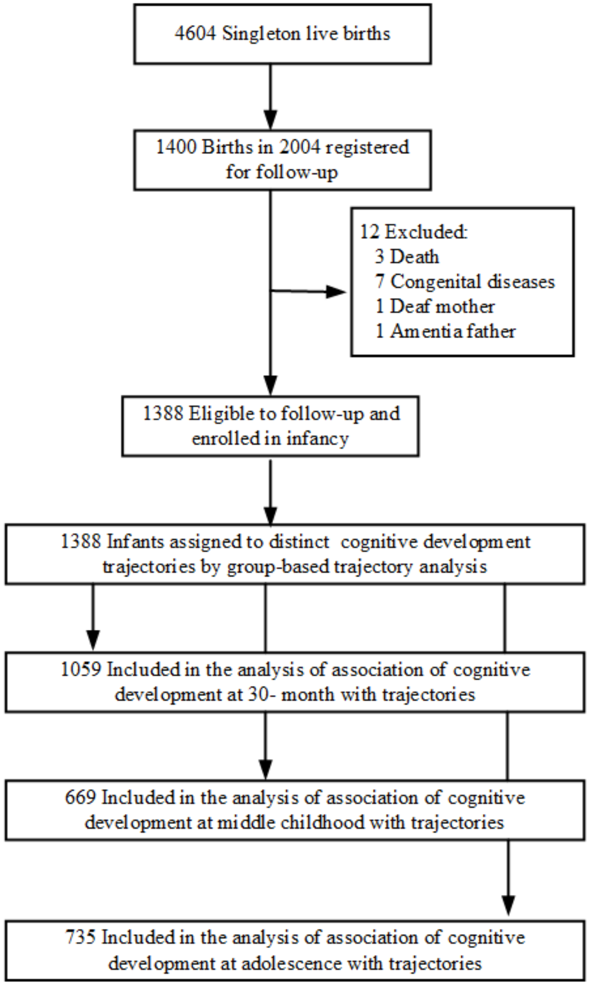

Supplementary Fig. S1 Flow chart of study participants
